# microRNA-130b downregulation potentiates chondrogenic differentiation of bone marrow mesenchymal stem cells by targeting SOX9

**DOI:** 10.1590/1414-431X202010345

**Published:** 2021-02-12

**Authors:** Penggui Zhang, Guangming Gao, Ziyu Zhou, Xuejun He

**Affiliations:** 1 The First Department of Orthopedics, Yulin First Hospital, Yulin, Shaanxi, China; 2 The Second Department of Orthopedics, the First People's Hospital of Xianyang, Xianyang, Shaanxi, China

**Keywords:** Osteoarthritis, miR-130b, SOX9, Chondrogenic differentiation, Bone marrow mesenchymal stem cells

## Abstract

Osteoarthritis (OA) is a chronic health condition. MicroRNAs (miRs) are critical in chondrocyte apoptosis in OA. We aimed to investigate the mechanism of miR-130b in OA progression. Bone marrow mesenchymal stem cells (BMSCs) and chondrocytes were first extracted. Chondrogenic differentiation of BMSCs was carried out and verified. Chondrocytes were stimulated with interleukin (IL)-1β to imitate OA condition *in vitro*. The effect of miR-130b on the viability, inflammation, apoptosis, and extracellular matrix of OA chondrocytes was studied. The target gene of miR-130b was predicted and verified. Rescue experiments were performed to further study the underlying downstream mechanism of miR-130b in OA. miR-130b first increased and drastically reduced during chondrogenic differentiation of BMSCs and in OA chondrocytes, respectively, while IL-1β stimulation resulted in increased miR-130b expression in chondrocytes. miR-130b inhibitor promoted chondrogenic differentiation of BMSCs and chondrocyte growth and inhibited the levels of inflammatory factors. miR-130b targeted SOX9. Overexpression of SOX9 facilitated BMSC chondrogenic differentiation and chondrocyte growth, while siRNA-SOX9 contributed to the opposite trends. Silencing of SOX9 significantly attenuated the pro-chondrogenic effects of miR-130b inhibitor on BMSCs. Overall, miR-130b inhibitor induced chondrogenic differentiation of BMSCs and chondrocyte growth by targeting SOX9.

## Introduction

Osteoarthritis (OA), a prevalent, progressive, and degenerative joint disorder, is characterized by articular cartilage degeneration, joint impairment, and abnormalities in other joint tissues, such as the synovial membrane and ligaments (1,2). OA is a common chronic health condition, leading to severe pain and disability (3), affecting more than 151 million individuals globally (4). It is believed that the increase in joint load due to weight gain is the cause of accelerated OA (5). OA is a key contributor to pain, stiffness, disability, poor quality of life, and limited activities of daily life (2). Unfortunately, OA is usually diagnosed via clinical and radiographic changes during the irreversible stages when the treatment of OA is largely palliative, only to slow the progression and reduce the pain (6). The exact pathogenesis of OA is still unclear, and preventive and modifying therapies are urgently needed (1). The exploration of novel biological markers and more effective approaches could lay a theoretical foundation for OA treatment.

MicroRNAs (miRs) are short single-stranded non-coding RNAs, whose altered expression has been described under various pathological conditions, including rheumatic and other autoimmune diseases, and in plasma and synovial fluid of patients with established OA (7). miRs, important regulators of chondrocyte growth, have been implicated in OA progression caused by chondrocyte injury (8). The dysregulation of miRs in tissue samples from OA patients has been found in previous studies, and their relevance to pathological progression has been also determined (9,10). Mesenchymal stem cells (MSCs) derived from bone marrow (BMSCs) are the best candidate for bone engineering and bone and cartilage homeostasis, mainly through their highly chondrogenic differentiation potentials (11). The correlation between the osteogenic differentiation of BMSCs with miRNAs has been underscored (12). Nevertheless, the involvement of miRs in the process of the chondrogenic differentiation of BMSCs awaits further investigation. miR-130b was highly expressed in osteolytic mice, promoting osteolysis and inflammation (13), which may lead to OA, while the specific role of miR-130b during the chondrogenic differentiation of BMSCs remains largely unknown. Moreover, OA mice exhibit decreased positive expression rate of SRY-box transcription factor 9 (SOX9) in cartilage tissues (14). In addition, miR-145 alteration induced profound changes in the phenotype of human articular chondrocytes partially through directly targeting SOX9 (15). Therefore, we postulated that miR-130b regulated the chondrogenic differentiation of BMSCs via interacting with SOX9. This study aimed to probe the possible mechanism of miR-130b in chondrogenic differentiation of BMSCs with the participation of SOX9.

## Material and Methods

### Isolation and culture of rat BMSCs and chondrocytes

Twelve healthy 12-week-old Sprague Dawley rats (6 males and 6 females) were obtained from the animal management center of Yulin First Hospital (China). All experiments obtained the ethical certification of the Animal Protection and Use Committee of Yulin First Hospital.

Six rats (3 males and 3 females) were randomly selected and euthanized with pentobarbital sodium (200 mg/kg) to separate BMSCs. Animal death was confirmed by observing the lack of heartbeat (excluding cardiac arrest) for 2-3 min, respiratory arrest, and lack of nerve reflex. Next, bilateral lower limbs of rats were extracted, the epiphyseal ends were removed and cut off, and the bone marrow in the marrow cavity was collected. BMSCs were dispersed into single cell suspension with phosphate buffer saline (PBS), and centrifuged at 1050 *g* for 10 min at 4°C. After removing the supernatant, cells were resuspended in F12/Dulbecco's modified Eagle’s medium (DMEM), seeded into a 25-cm^2^ flask, and cultured in 5% CO_2_ at 37°C. The cell concentration of Petri dishes was about 6.37×10^6^ cells/mL. The media were refreshed every 2 days. After cell confluency, the cells were detached with 0.25% trypsin, and subcultured at 1:2. Cells after passage 3 were used for subsequent experiments. Cell morphology and growth were observed using an inverted microscope (AZ100; Nikon Corporation, Japan).

The remaining 6 rats (3 males and 3 females) were euthanized using pentobarbital sodium (200 mg/kg) to separate chondrocytes. The euthanasia was verified as above. Bilateral lower limbs, epiphyseal ends, and bone marrow were treated as above. Chondrocytes were isolated from the femoral condyle and tibial plateau of the rats. The articular cartilage of rats was cut into small pieces and detached with 0.25% trypsin (Invitrogen, USA) for 30 min, and then detached with 0.3% type II collagenase (Invitrogen) at 37°C for 4 h. The cells were suspended in DMEM (Invitrogen) with 10% fetal bovine serum (FBS, Thermo Fisher, China), 100 U/mL penicillin and 100 U/mL streptomycin in moist air at 37°C and 5% CO_2_. The cell concentration of Petri dishes was approximately 1.32×10^7^ cells/mL. The primary chondrocytes with 80% confluence were used in the following study.

### Identification and differentiation of BMSCs

The specific cell surface markers of BMSCs were identified by flow cytometry. In short, BMSCs after passage three were diluted to 3×10^6^ cells/mL with PBS containing 0.1% bovine serum albumin. Resuspended cells were cultured with 5 μL fluorescein isothiocyanate-labeled anti-rat CD29, CD44, CD73, CD90, and CD105 and phycoerythrin-conjugated anti-rat CD34, and CD45 (all from BD, Biosciences, USA) in the dark at 4°C for 30 min (16). The labeled cells were analyzed on a FACS Aria machine (BD Biosciences).

After differentiation of BMSCs (1×10^6^) in rat BMSC osteogenic medium (CP1207, Shenzhen Weitong Biotechnology Co., Ltd., China) for three weeks, calcium deposits in cells were identified using alizarin red staining solution. Similarly, after differentiation of 1×10^6^ BMSCs in adipogenic differentiation medium (CP1216, Shenzhen Weitong Biotechnology Co., Ltd.) for two weeks, lipid production was identified by oil red O staining.

For chondrogenic differentiation, 1×10^6^ BMSCs were suspended and centrifuged (300 *g*; 4 min; 37°C) to form cell clusters in a V-shaped polypropylene tube at 37°C with 5% CO_2_. The cells were supplemented with cartilage-inducing solution (containing 50 mg/L ascorbic acid, 1% FBS, 100 nM dexamethasone, 100 mg/L sodium pyruvate, 1.0% indomethacin, 40 mg/L proline, and low-glucose DMEM) to make the cell mass non-dispersive. The media were renewed every 3 days, and the cartilage mass was formed after 3 weeks of induction in the V-type polypropylene culture tube.

### Toluidine blue staining

After BMSC culturing for 21 days, cartilage formation was identified by toluidine blue staining. The cell mass was fixed for 24 h in 4% paraformaldehyde, dehydrated in ethanol gradient (70-96%), and embedded in paraffin. The 10-µm sections were stained for 20 min with 0.5% toluidine blue (Sigma-Aldrich, Germany) solution and the stained cells were then observed using a phase contrast microscope (Olympus, Japan).

### Immunofluorescence staining

Cartilage formation markers of BMSCs were identified by immunofluorescence staining after 21 days of chondrogenic differentiation. Cells were fixed with paraformaldehyde and blocked for 1 h with 1% BSA. The cells were incubated overnight with the primary antibodies against Collagen II (Col II, ab34712) or Collagen X (ab58632) at 4°C and then incubated with the secondary goat anti-rabbit antibody IgG H&L (Alexa Fluor^®^594, ab150080 and Alexa Fluor^®^ 488, ab150077) (all from Abcam, UK) at room temperature to avoid light exposure. 4′,6-diamidino-2-phenylindole (DAPI) was used for the nucleus staining.

### Cell transfection

Chondrocytes were treated with interleukin (IL)-1β (5 ng/mL, Peprotech, USA) or PBS. miR-130b mimic/inhibitor, siRNA-SOX9, pcDNA-SOX9, and their respective controls used for transfection of chondrocytes or BMSCs before differentiation were designed and synthesized by Shanghai GenePharma Co., Ltd. (China). According to the manufacturer's instructions, Lipofectamine 2000 (Invitrogen) was applied for transfection.

### Quantitative real-time polymerase chain reaction (RT-qPCR)

Total RNA was extracted with a TRIzol kit (Invitrogen) and reversely transcribed into cDNA using the High-Capacity RNA-to-cDNA™ kit (Thermo Fisher). SYBR Green PCR Master Mix kit (TaKaRa, Japan) was utilized to measure Sox9 expression. GAPDH was the internal reference of Sox9. TaqMan miRNA assay (Applied Biosystems, USA) was utilized to measure miR-130b expression normalized to U6. The multiple change was calculated by 2^-△△Ct^ method. The primer sequences are demonstrated in Table 1.

**Table 1:** Primer sequences for RT-qPCR.

Gene	Forward primer (5′-3′)	Reverse primer (5′-3′)
miR-130b	ACTCTTTCCCTGTTGCACT	GAACATGTCTGCGTATCTC
SOX9	AGGAAGCTGGCAGACCAGTA	ACGAAGGGTCTCTTCTCGCT
IL-6	AGGAGTGGCTAAGGACCAAGACC	TGCCGAGTAGACCTCATAGTGACC
TNF-α	TCTTCTCATTCCTGCTCGTGG	TGATGAGAGGGAGCCCATTTG
GAPDH	ACCTCAACTACATGGTCTAC	TTGTCATTGAGAGCAATCC
U6	CTCGCTTCGGCAGCACA	AACGCTTCACGAATTTGCGT

miR-130b: microRNA; SOX9: SRY-box transcription factor 9; IL: interleukin; TNF-α: tumor necrosis factor alpha; GAPDH: glyceraldehyde-3-phosphate dehydrogenase.

### Western blot analysis

Protein was extracted using lysis buffer (Thermo Fisher), and the concentration was evaluated using a bicinchoninic acid kit (Pierce, USA). Equal amounts of protein were taken from each sample, and transferring was performed with 10% SDS-PAGE (Bio-Rad, USA) using the semi-transfer method after the protein was mixed with loading buffer. The membrane was probed with primary antibodies to Col II (1:10000, ab34712, Abcam), aggrecan (1:100, ab36861, Abcam), Bax (1:1000, #14796, Cell Signaling Technology, USA), Bcl-2 (1:2000, #33-6100, Thermo Fisher), and β-actin (1:5000, ab119716, Abcam) overnight at 4°C. After tris-buffered saline-Tween (TBST) washing, the membrane was incubated with secondary antibodies goat anti-rabbit IgG H&L (HRP) (1:50000, ab205718, Abcam) and goat anti-mouse IgG H&L (HRP) (1:10000, ab205719, Abcam) at room temperature for 2 h, followed by one TBST washing. Proteins were detected by chemiluminescence (Millipore, USA).

### 3-(4,5-dimethylthiazol-2-yl)-2,5-diphenyltetrazolium bromide (MTT) assay

The chondrocytes were seeded into 96-well plates at 6000 cells per well for 24 h. Then, 100 μL MTT solution (1 mg/mL, Sigma-Aldrich) was added to each well. Absorbance at 490 nm was recorded by a SPECTROMAX M5 microplate reader (Molecular Devices, USA).

### Colony formation assay

After detachment with 0.25% trypsin, the treated chondrocytes were planted onto 6-well microplates at 500 cells/well for 2-3 weeks at 37°C with 5% CO_2_, and media were renewed every 3 days. The cells were then stained with crystal violet (Beijing Solarbio Science & Technology Co., Ltd., China) and observed under an Axiovert 200 inverted microscopy (Zeiss, Germany). Five fields were randomly chosen to count the colonies, and the results were averaged.

### Cell apoptosis assays

TUNEL method was applied to measure apoptosis of chondrocytes (1×10^6^). The cell nuclei were stained with DAPI (Shanghai Beyotime Biotechnology Co., Ltd., China), and cells were stained with TUNEL using a TUNEL fluorescent kit (Roche, Switzerland). The number of cells with TUNEL-positive staining was calculated using laser scanning confocal microscopy (SP8, Leica, Japan) to determine the frequency of apoptotic cells.

For flow cytometry, apoptosis was detected by annexin V-FITC cell apoptosis detection kits (Bestbio, China). A total of 1×10^5^ cells/well grew on the 6-well plate. The cells were cultured for 48 h after transfection, then detached with trypsin and collected. After PBS washing, cells were resuspended in 1× binding buffer at 1×10^5^ cells/mL. Then, 5 μL fluorescein isothiocyanate (FITC) annexin-V and 5 μL propidium iodide were added to 100 μL cell suspension, and the samples were incubated for 15 min in the dark. After that, 400 μL 1× binding buffer was added. Cell quest software (BD Biosciences) was used to analyze apoptosis on the FACS Calibur cytometer (BD Biosciences).

### Dual-luciferase reporter gene assay

The potential binding sites between miR-130b and SOX9 were predicted from Starbase (http://starbase.sysu.edu.cn/). The SOX9 fragment containing the putative miR-130b binding site was subcloned into pmirGLO vector (Promega, USA) and named wild type (WT). Then, the predicted miR-130b binding site was mutated to make a mutant (MT). Two reported plasmids were co-transfected with miR-130b mimic into 293T cells (purchased from ATCC). After 48 h of transfection, luciferase activity was measured by dual-luciferase reporting systems (Promega).

### Statistical analysis

SPSS 22.0 statistical software (IBM Corp., USA) was utilized to process the data. All data are reported as means±SD of at least 3 independent experiments. Data with normal distribution were examined using the Kolmogorov-Smirnov method. The comparisons between two groups were done by the *t*-test and those among multiple groups were conducted by the one-way or two-way analysis of variance (ANOVA). Tukey's multiple comparison test was used for *post hoc*-test. A probability value of P<0.05 indicated the difference was statistically significant.

## Results

### miR-130b was downregulated in chondrogenic differentiation and upregulated in OA chondrocytes

**Figure 1:**
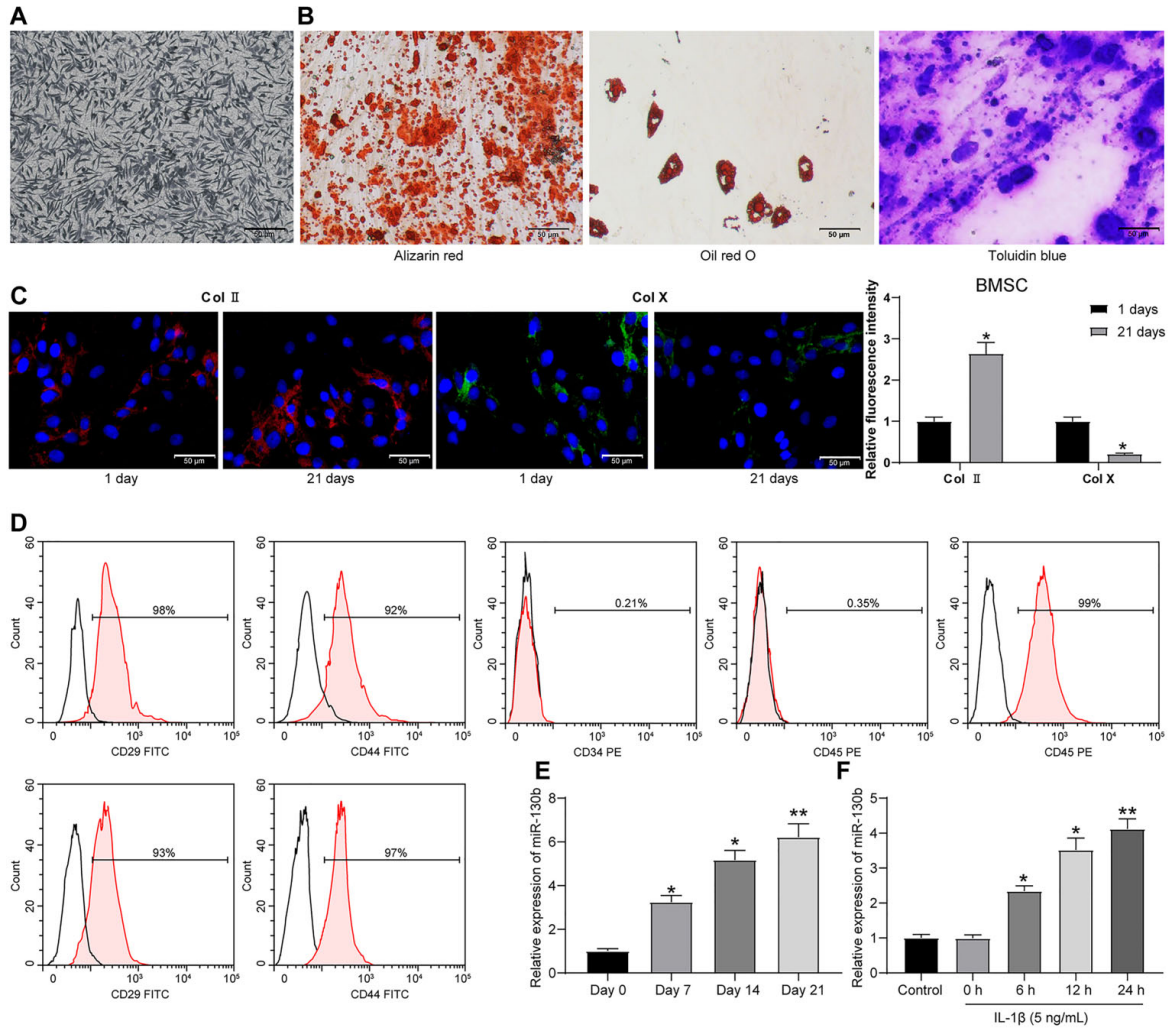
miR-130b is reduced in chondrogenic differentiation and enhanced in osteoarthritis chondrocytes. **A**, Morphology of bone marrow mesenchymal stem cells (BMSCs) observed by electron microscope. **B**, Alizarin red staining, oil red O staining, and toluidine blue staining were used to measure the osteogenic, lipogenic, and chondrogenic abilities of BMSCs (scale bar, 50 μm). **C**, Col II and Col X expression in BMSCs determined by immunofluorescence assay (scale bar, 50 �m). **D**, Flow cytometry identified BMSCs. **E**, RT-qPCR measured miR-130b expression in chondrogenic differentiation of BMSCs. **F**, RT-qPCR measured miR-130b expression in interleukin-1β-treated chondrocytes. The data in panels **E** and **F** were analyzed using one-way ANOVA, and the data in panel **C** were analyzed using two-way ANOVA. *P<0.05, **P<0.01 compared to control. Experiments were repeated 3 times independently.

Cell morphology of BMSCs was observed by an inverted microscope (Figure 1A). The cells had typical spindle shape in spiral or clustered growth, which are the typical features of BMSCs. Then, the differentiation abilities of MSCs were identified by alizarin red, oil red O, and toluidine blue (Figure 1B). After 21 days of osteogenesis induction, many red spots were observed in the cells by alizarin red staining. These are calcified nodules containing a small number of mineral deposits. After 14 days of adipogenesis induction, dark red deposits were observed in the cells by oil red O staining, which were the deposition of lipids, appearing as droplets or beads. After chondrogenic induction for 14 days, the synthesis of extracellular matrix was observed. Chondrogenic culture significantly promoted Col II expression and inhibited Col X expression (Figure 1C). Flow cytometry analysis showed that CD29 (98%), CD44 (92%), CD73 (99%), CD90 (93%), and CD105 (97%) were positive, and CD34 (0.21%) and CD45 (0.35%) were negative (Figure 1D).

miR-130b expression was measured by RT-qPCR on days 0, 7, 14, and 21 after chondrogenic differentiation of BMSCs. The expression of miR-130b increased significantly at the 7th day, then decreased rapidly, and the expression of miR-130b reached the lowest level on the 21st day (Figure 1E). After treatment with 5 ng/mL IL-1β for 0, 6, 12, and 24 h, IL-1β significantly increased miR-130b expression in chondrocytes in a time-dependent manner compared with untreated control chondrocytes (Figure 1F).

### Inhibition of miR-130b promoted chondrogenic differentiation of BMSCs and chondrocyte growth

**Figure 2:**
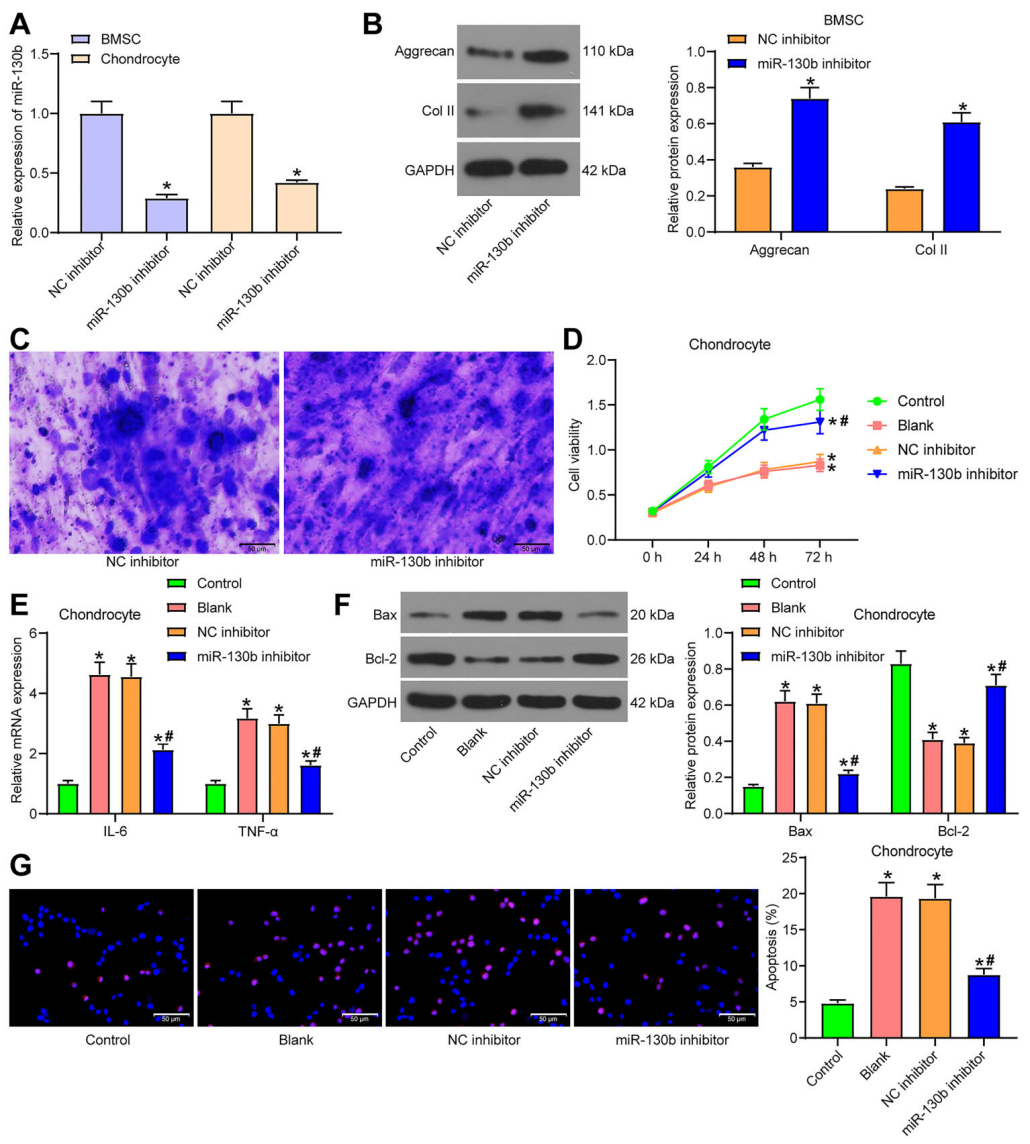
Inhibition of miR-130b promotes chondrogenic differentiation of bone marrow mesenchymal stem cells (BMSCs) and chondrocyte growth. **A**, The transfection efficiency of miR-130b inhibitor was verified by RT-qPCR. **B**, Western blot analysis measured the expression of two cartilage markers in BMSCs. **C**, The synthesis of extracellular matrix during chondrogenesis of BMSCs was examined by toluidine blue staining (scale bar, 50 μm). **D**, MTT method was used to measure the viability of interleukin (IL)-1β-stimulated chondrocytes. **E**, IL-6 and tumor necrosis factor (TNF)-α expression in IL-1β-treated chondrocytes was determined by RT-qPCR. **F**, Levels of apoptosis-related proteins in IL-1β-treated chondrocytes were measured by western blot analysis. **G**, Apoptosis rate in IL-1β-treated chondrocytes was measured by TUNEL assay (scale bar, 50 �m). *P<0.05, compared to control; ^#^P<0.05 compared to the NC inhibitor group. The data in panels **A** and **B** were analyzed using one-way ANOVA, and the data in panels **D**, **E**, **F**, and **G** were analyzed using two-way ANOVA. Experiments were repeated 3 times independently. NC: negative control.

miR-130b inhibitor and its control were transfected into BMSCs and chondrocytes, and RT-qPCR confirmed that miR-130b inhibitor reduced the expression of miR-130b in the BMSCs by about 3.4 times, while it reduced the expression of miR-130b in the chondrocytes by about 2.3 times (Figure 2A). miR-130b inhibitor and its control were transfected into MSCs and cultured for 21 days to assess the levels of Col II and aggrecan. miR-130b inhibitor significantly elevated the levels of the two cartilage markers (Figure 2B). Toluidine blue staining indicated that miR-130b inhibitor significantly promoted the synthesis of extracellular matrix (Figure 2C).

miR-130b inhibitor and its control were transfected into IL-1β-treated chondrocytes. The untreated chondrocytes were used as the controls. The IL-1β-treated chondrocytes that were not transfected with plasmid were the blank group. Using MTT assay, we found that IL-1β treatment significantly inhibited the viability of cells, while miR-130b inhibitor partially restored the viability of cells (Figure 2D). The expression of IL-6 and tumor necrosis factor (TNF)-α was measured by RT-qPCR. IL-1β significantly increased the release of inflammatory factors in cells, and miR-130b inhibitor partially mitigated this trend (Figure 2E). Western blot analysis (Figure 2F) and TUNEL staining (Figure 2G) indicated that IL-1β enhanced Bax expression and inhibited Bcl-2 expression significantly, and promoted the apoptosis rate of chondrocytes, while miR-130b inhibitor partially abrogated the effect of IL-1β treatment on cell apoptosis.

### miR-130b targeted SOX9

**Figure 3:**
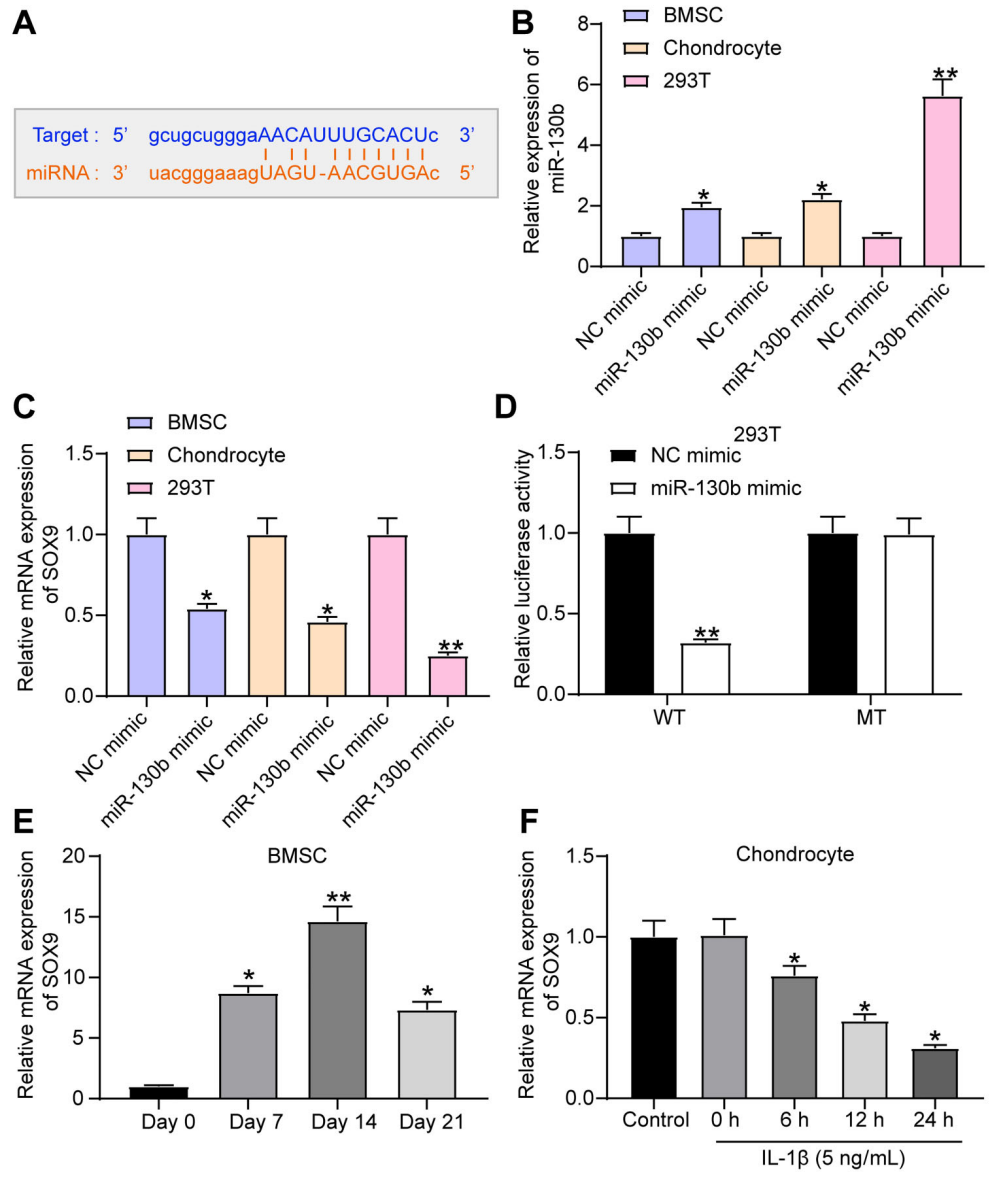
miR-130b targeted SOX9. **A**, Potential binding site of miR-130b to SOX9. **B**, RT-qPCR measured the transfection efficiency of miR-130b mimic. **C**, RT-qPCR measured the effect of miR-130b mimic on SOX9 expression. **D**, Luciferase activity of 293T cells co-transfected with wild type (WT) and mutant (MUT)-SOX9 reporter plasmids, miR-130b mimic, and their controls measured by dual-luciferase reporter assay. **E**, SOX9 expression during chondrogenic differentiation of bone marrow mesenchymal stem cells (BMSCs) detected by RT-qPCR. **F**, RT-qPCR detected SOX9 expression in chondrocytes. *P<0.05, **P<0.01 compared to control. Data in panels **B**, **C**, **E**, and **F** were analyzed using one-way ANOVA and data in panel **D** were analyzed using two-way ANOVA. Experiments were repeated 3 times independently. NC: negative control.

We predicted that miR-130b has a potential binding site with SOX9 through Starbase (http://starbase.sysu.edu.cn/) (Figure 3A). We transfected miR-130b mimic and its control into BMSCs, chondrocytes, and 293T cells, and RT-qPCR verified the effective transfection (Figure 3B). miR-130b mimic significantly reduced SOX9 expression in BMSCs and chondrocytes (Figure 3C). The direct binding relationship between them was verified by the dual-luciferase report gene assay in 293T cells. The SOX9-WT report plasmid with potential binding sites to miR-130b and SOX9-MT report plasmid with mutation were constructed. The two plasmids and miR-130b mimic were co-transfected into 293T cells. miR-130b mimic significantly decreased the luciferase activity of SOX9-WT but had no significant effect on SOX9-MT (Figure 3D). SOX9 expression was measured by RT-qPCR on days 0, 7, 14, and 21 of BMSC chondrogenic differentiation. SOX9 expression increased slowly and then decreased, but the final expression was higher than that at the early stage (Figure 3E). After treatment with 5 ng/mL IL-1β at 0, 6, 12, or 24 h, IL-1β significantly inhibited SOX9 expression in a time-dependent manner compared with untreated controls (Figure 3F).

### Overexpression of SOX9 promoted chondrogenic differentiation of BMSCs and chondrocyte growth

**Figure 4:**
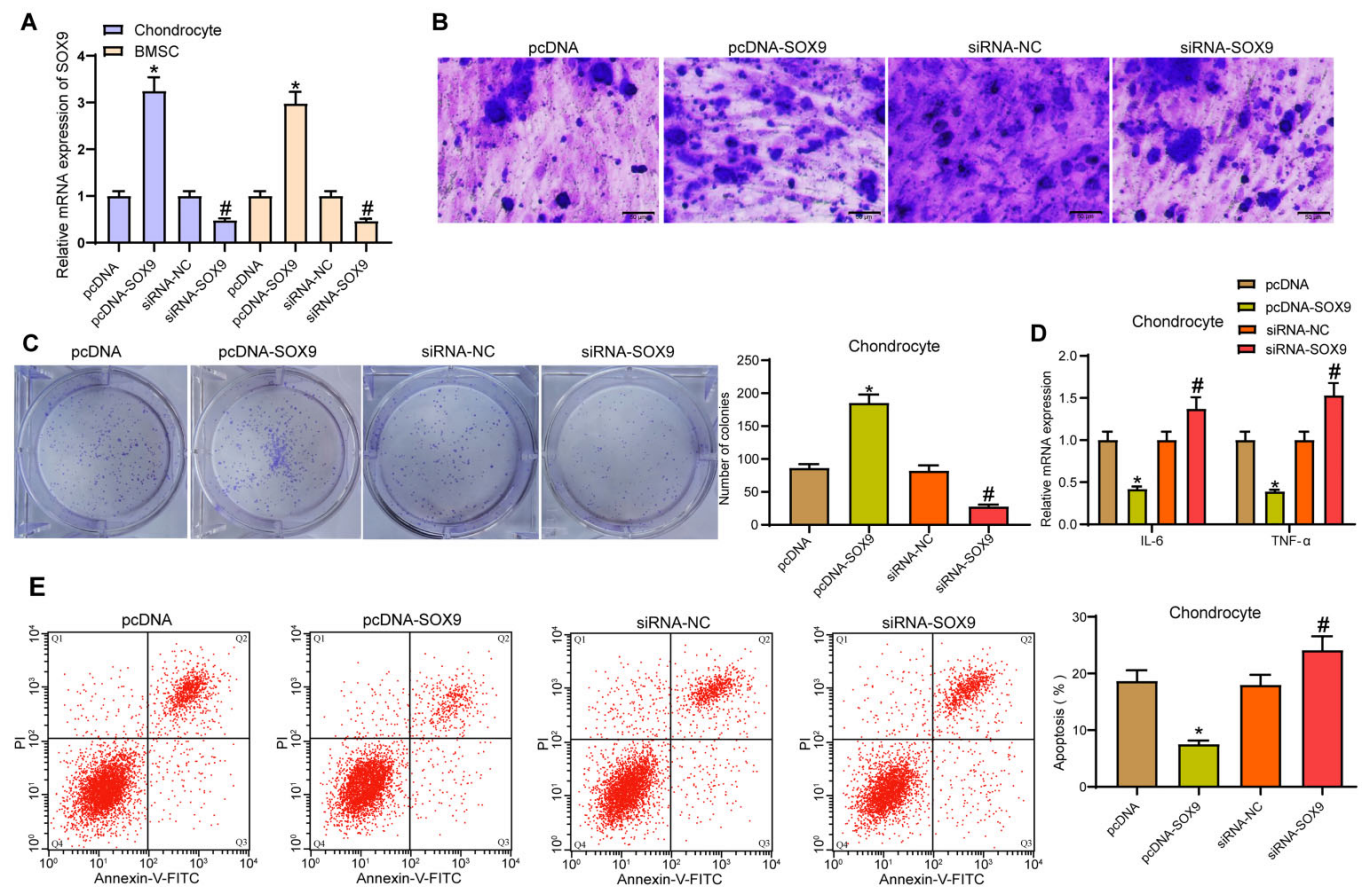
Overexpression of SOX9 promotes chondrogenic differentiation of bone marrow mesenchymal stem cells (BMSCs) and chondrocyte growth. **A**, Transfection efficiency measured by RT-qPCR. **B**, Toluidine blue staining detected the effect of pcDNA-SOX9 and siRNA-SOX9 on the synthesis of extracellular matrix in the process of cartilage differentiation of BMSCs (scale bar, 50 μm). **C**, Effect of pcDNA-SOX9 and siRNA-SOX9 on the proliferation of interleukin (IL)-1β-treated chondrocytes measured by colony formation assay. **D**, RT-qPCR measured the effects of pcDNA-SOX9 and siRNA-SOX9 on the expression of IL-6 and TNF-α. **E**, Effect of pcDNA-SOX9 and siRNA-SOX9 on apoptosis of IL-1β-treated chondrocytes measured by flow cytometry. Data in panels **A**, **C**, and **E** were processed using one-way ANOVA, and data in panel **D** were analyzed using two-way ANOVA. *P<0.05 compared to cells transfected with pcDNA; ^#^P<0.05 compared to cells transfected with siRNA-NC. Experiments were repeated 3 times independently.

pcDNA-SOX9, siRNA-SOX9, and their controls were transfected into BMSCs and chondrocytes, and the transfection efficiency was measured by RT-qPCR (Figure 4A).

BMSCs transfected with pcDNA-SOX9, siRNA-SOX9, and their controls were cultured for 21 days. Toluidine blue staining indicated that pcDNA-SOX9 significantly promoted the synthesis of extracellular matrix, while siRNA-SOX9 inhibited the synthesis of extracellular matrix (Figure 4B).

pcDNA-SOX9, siRNA-SOX9, and their controls were transfected into IL-1β-treated chondrocytes. Colony formation assay showed that pcDNA-SOX9 significantly promoted cell proliferation, whereas siRNA-SOX9 reduced the colonies formed (Figure 4C). By RT-qPCR, we found that pcDNA-SOX9 significantly inhibited the expression of IL-6 and TNF-α, while siRNA-SOX9 exerted a pro-inflammatory function (Figure 4D). Flow cytometry showed that pcDNA-SOX9 significantly inhibited the apoptosis of chondrocytes, while siRNA-SOX9 enhanced chondrocyte apoptosis (Figure 4E).

### SOX9 silencing weakened the repressive role of miR-130b inhibitor on chondrogenic differentiation of BMSCs and chondrocyte growth

**Figure 5:**
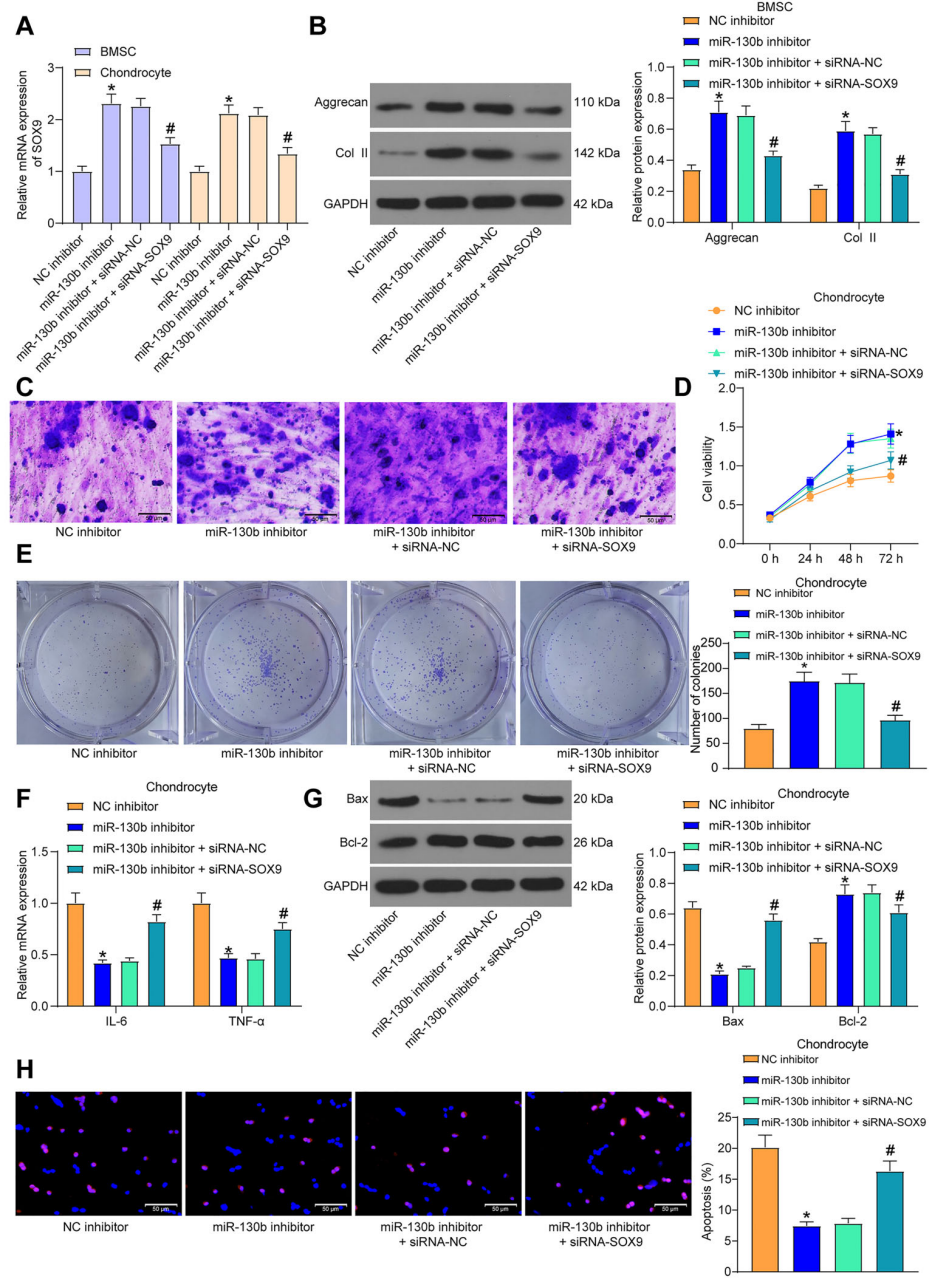
SOX9 silencing weakened the inhibition of miR-130b inhibitor on chondrogenic differentiation of bone marrow mesenchymal stem cells (BMSCs) and chondrocyte growth. **A**, Expression of SOX9 in BMSCs and chondrocytes was measured by RT-qPCR. **B**. Western blot analysis measured the protein level of aggrecan and Col II in BMSCs. **C**, Toluidine blue staining indicated the extracellular matrix synthesis of BMSCs (scale bar, 50 μm). **D**, MTT method measured the viability of chondrocytes. **E**, Colony formation ability of chondrocytes. **F**, RT-qPCR measured the expression of interleukin (IL)-6 and tumor necrosis factor (TNF)-α in chondrocytes. **G**, Western blot analysis measured the expression of apoptosis-related factors in chondrocytes. **H**, TUNEL staining examined the apoptosis rate of chondrocytes (scale bar, 50 �m). The data in panels **A**, **E**, and **H** were analyzed using one-way ANOVA, and the data in panels **B**, **D**, **F,** and **G** were analyzed using two-way ANOVA. *P<0.05 compared to cells transfected with NC inhibitor; ^#^P<0.05 compared to cells transfected with miR-130b inhibitor + siRNA-NC. Experiments were repeated 3 times independently. NC: negative control.

miR-130b inhibitor, miR-130b inhibitor + siRNA-SOX9, and their respective controls were transfected into BMSCs and chondrocytes. SOX9 expression was verified by RT-qPCR (Figure 5A). The promoting effect of miR-130b inhibitor on Col II and aggrecan was partially abrogated by siRNA-SOX9 (Figure 5B). Toluidine blue staining indicated that the promoting effect of miR-130b inhibitor on extracellular matrix synthesis was partially mitigated by siRNA-SOX9 (Figure 5C).

After the transfected cells were treated with IL-1β, MTT (Figure 5D) and colony formation assays (Figure 5E) showed that the effects of miR-130b inhibitor on viability and colony formation abilities of chondrocytes were partially antagonized by siRNA-SOX9. RT-qPCR and western blot indicated that siRNA-SOX9 promoted the levels of these pro-inflammatory and pro-apoptotic factors in the presence of miR-130b inhibitor (Figure 5F and G). Flow cytometry also indicated that siRNA-SOX9 partially reversed the anti-apoptotic role of miR-130b inhibitor in chondrocytes (Figure 5H).

## Discussion

The current approaches for OA are mainly painkiller drugs, surgery, and exercise, which are either just treating symptoms, or present adverse effects, or time-consuming (17). miRs altering the homeostasis of articular cartilage have emerged as a research focus for OA biomarkers and therapeutic targets (18). We verified in this study that miR-130b inhibitor potentiated chondrogenic differentiation of BMSCs and chondrocyte growth by targeting SOX9.

Several studies have revealed the implication of miRs in the chondrogenic differentiation of BMSCs in the development of OA (19,20). miR-130b expression increased firstly over chondrogenic differentiation of BMSCs and sharply reduced and upregulated in OA chondrocytes. miR-130b presents high expression in umbilical cord MSCs, adipose tissue MSCs, and BMSCs (21). Moreover, has-miR-130b is overexpressed in osteo-differentiated MSCs (22). We believe that miR-130b expression showed a transient increase during differentiation culture and decreased with chondrogenic differentiation. Additionally, IL-1β regulates miR-675-3p expression in a time- and dose-dependent manner in chondrocytes (23). In the current study, IL-1β significantly increased miR-130b expression in a time-dependent manner. In the indirect co-culture system, chondrocyte-derived exosomal miR-8485 induces differentiation of BMSCs to chondrocytes (24). miRs have been documented to be linked to cartilage imbalance in response to IL-1β stimulation (25). Therefore, we concluded that miR-130b inhibitor showed fluctuated expression during chondrogenic differentiation of BMSCs and was enhanced by IL-1β stimulation in chondrocytes.

Chondrocytes are unique cell components of adult articular cartilage, which maintain extracellular matrix components and are activated in OA (26). Col II and aggrecan are unique chondrogenic markers (27). miR-130b inhibitor significantly promoted the expression Col II and aggrecan and promoted the synthesis of extracellular matrix. Collagen and ILs involved in cartilage remodeling and collagen degradation are predicted as potential targets of dysregulated miRs during OA (28). In our study, IL-1β treatment significantly inhibited the viability of cells and increased the expression of inflammatory factors in cells, while miR-130b inhibitor partially reversed these trends. The inflammatory mediator IL-1β promotes extracellular matrix degradation and chondrocyte apoptosis and causes cartilage damage (23). Inflammatory cytokines maintain the balance of extracellular matrix and matrix-degrading proteinases (29). During OA progression, the activated chondrocytes facilitate apoptosis and are pro-inflammatory, leading to loss of extracellular matrix (30). IL-1β increased Bax expression, inhibited Bcl-2 expression, and promoted the apoptosis rate of chondrocytes, while miR-130b inhibitor partially compromised the effect of IL-1β treatment on cell apoptosis. Similarly, miR-203a inhibition decreases IL-1β-induced reduction in cell viability, apoptosis, inflammation, and extracellular matrix metabolic imbalance (30). Overall, inhibition of miR-130b promoted cartilage differentiation of BMSCs and chondrocyte growth.

Bioinformatics analysis in a previous study reveals that OA pathology is associated miR-mRNA target interactions (18). Through Starbase software and dual-luciferase reporter gene assay, we discovered that miR-130b targeted SOX9. SOX9 is important in MSC differentiation (31) and interactions between miR and SOX9 in MSC differentiation have been reported. For example, SOX9-regulated miR-574-3p inhibits MSC chondrogenic differentiation (32). SOX9 is essential in chondrocyte development by regulating the chondrocyte-specific genes, including Col II and aggrecan (33). Our observations indicated that the expression of SOX9 was increased from day 0 to day 14 and then decreased from day 14 of the chondrogenic differentiation of BMSCs to day 21 and significantly reduced in OA chondrocytes. SOX9 declines upon chondrogenic differentiation in cells exposed to IL-1β (34). SOX9 expression was reduced in OA samples compared with the age-matched controls (35,36). By contrast, IL-1β treatment alone decreases SOX9, Col II, and aggrecan (37).

Moreover, pcDNA-SOX9 markedly promotes the synthesis of extracellular matrix, inhibits levels of IL-6 and TNF-α and apoptosis of chondrocytes. SOX9, like Col II and aggrecan, is a member of extracellular matrix-related genes (38). Inflammatory cytokines maintain the balance between the synthesis and catabolism of extracellular matrix proteins (29). The promoting effect of miR-130b inhibitor on Col II, aggrecan, and extracellular matrix synthesis was partially reversed by siRNA-SOX9. The chondrogenic differentiation ability of MSCs was greatly inhibited after SOX9 interference (39). SOX9 upregulation markedly repressed IL-1β-stimulated apoptosis and inflammation in chondrocytes, and alleviated OA symptoms in mice (40).

**Figure 6:**
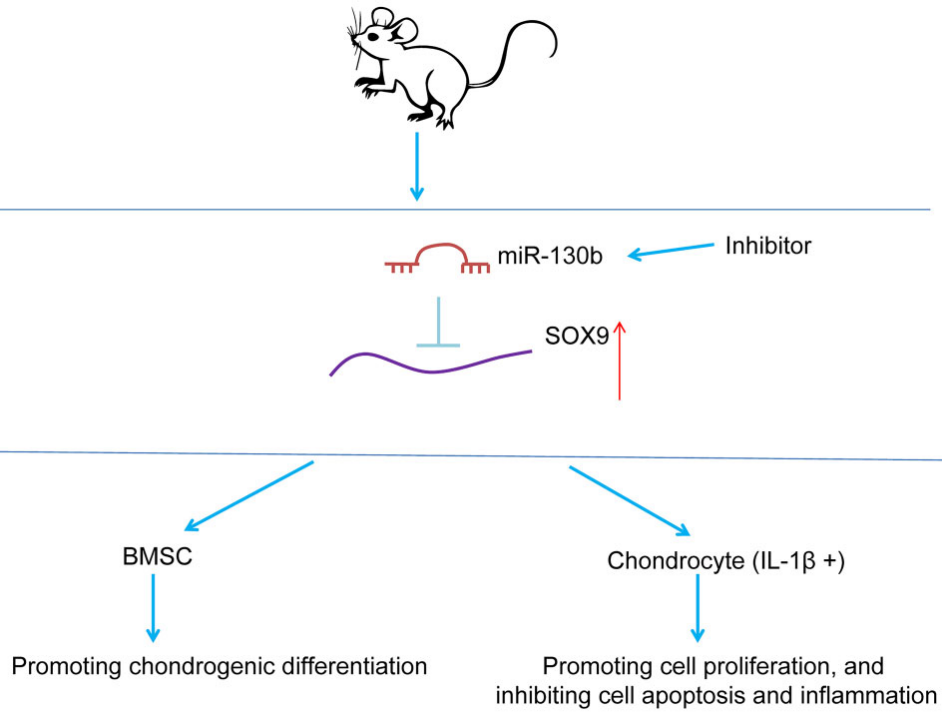
Experimental mechanism diagram. The inhibition of miR-130b promotes the chondrogenic differentiation of bone marrow mesenchymal stem cells (BMSCs) and chondrocyte growth by targeting SOX9.

In summary, we highlighted that miR-130b inhibitor expedited chondrogenic differentiation of BMSCs and chondrocyte growth by targeting SOX9 (Figure 6). Due to the limitations of time and funding, we did not perform an exhaustive investigation of pathways downstream of SOX9. The study of OA-related miRs is still in its infancy, and much work is needed to transform it into clinical application.
