# **Erratum notice for:** “microRNA-130b downregulation potentiates chondrogenic differentiation of bone marrow mesenchymal stem cells by targeting SOX9” [Braz J Med Biol Res 2021;54(4): e10345]

**DOI:** 10.1590/1414-431X2023e10345erratum

**Published:** 2023-04-14

Penggui Zhang^1^https://orcid.org/0000-0002-8930-4902, Guangming Gao^1^https://orcid.org/0000-0002-7467-5903, Ziyu Zhou^1^https://orcid.org/0000-0002-9648-3785, and Xuejun He^2^https://orcid.org/0000-0002-7036-7938

^1^The First Department of Orthopedics, Yulin First Hospital, Yulin, Shaanxi, China

^2^The Second Department of Orthopedics, the First People’s Hospital of Xianyang, Xianyang, Shaanxi, China

Correspondence: Xuejun He: <xuejunhe022701@163.com>

The authors notified the Editors of the Brazilian Journal of Medical and Biological Research that high magnification fields were erroneously shown in the Results section in Figure 1B, Figure 2C, Figure 4B, and Figure 5C for the quantification of toluidine blue staining. Because there were no differences between the two groups in the original Figure 2C, the toluidine blue staining images were incorrectly displayed. Therefore, all of the toluidine blue panels were modified to images without magnification, which were selected because they were more representative of the results. The images of Alizarin red staining and oil red O staining in Figure 1B were also modified to be more representative of the results. The statistical tests used for analyses were modified in the legend of Figure 2 panels B and G.

The correct Figures 1B, 2C, 4B, 5C are below:

**Figure 1:**
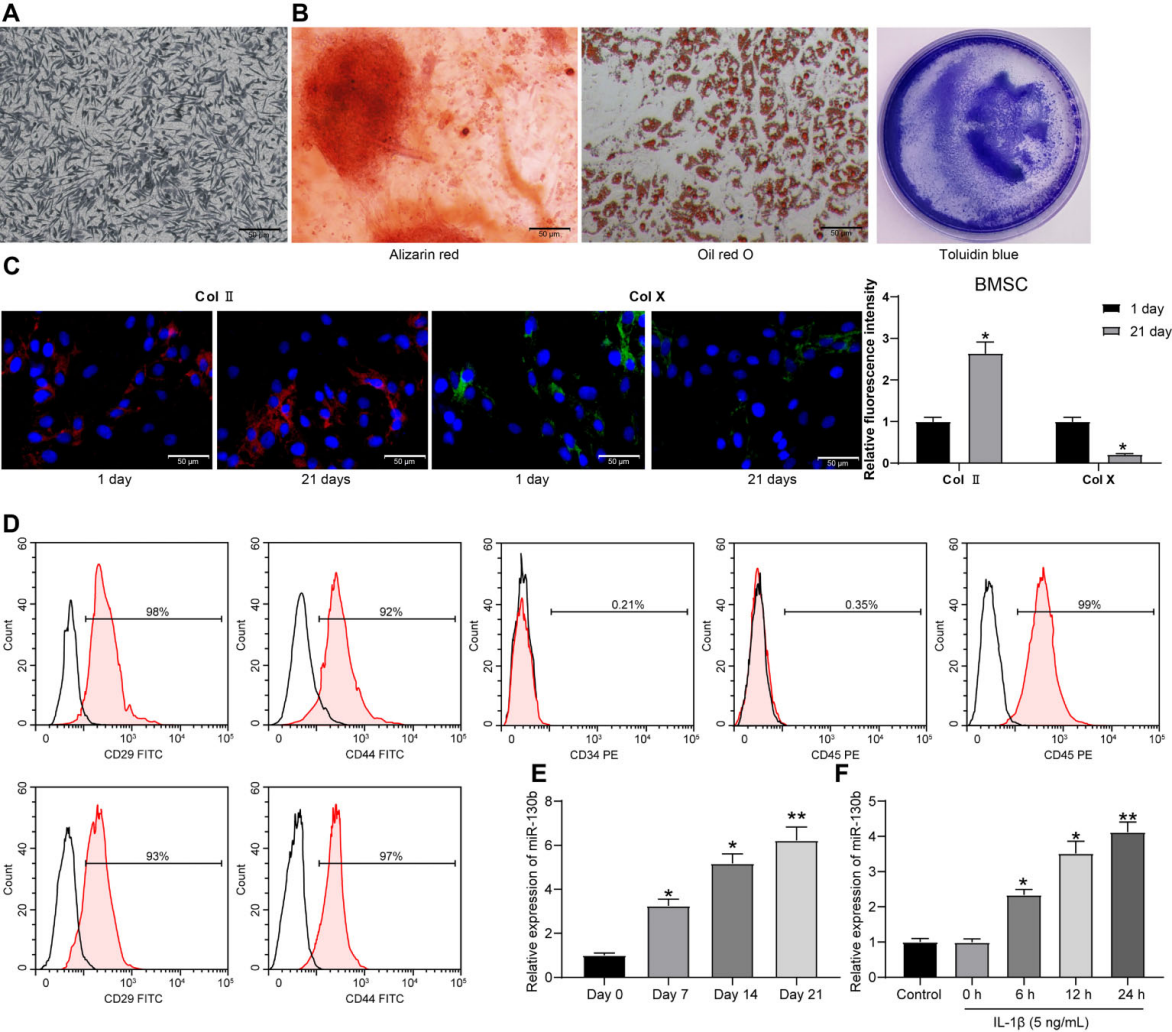
miR-130b is reduced in chondrogenic differentiation and enhanced in osteoarthritis chondrocytes. **A**, Morphology of bone marrow mesenchymal stem cells (BMSCs) observed by electron microscope (scale bar 50 μm). **B**, Alizarin red staining (scale bar 50 μm), oil red O staining (scale bar 50 μm), and toluidine blue staining were used to measure the osteogenic, lipogenic, and chondrogenic abilities of BMSCs. **C**, Col II and Col X expression in BMSCs determined by immunofluorescence assay (scale bar, 50 μm). **D**, Flow cytometry identified BMSCs. **E**, RT-qPCR measured miR-130b expression in chondrogenic differentiation of BMSCs. **F**, RT-qPCR measured miR-130b expression in interleukin-1β-treated chondrocytes. The data in panels E and F were analyzed using one-way ANOVA, and the data in panel C were analyzed using two-way ANOVA. *P<0.05, **P<0.01 compared to control. Experiments were repeated 3 times independently.

**Figure 2:**
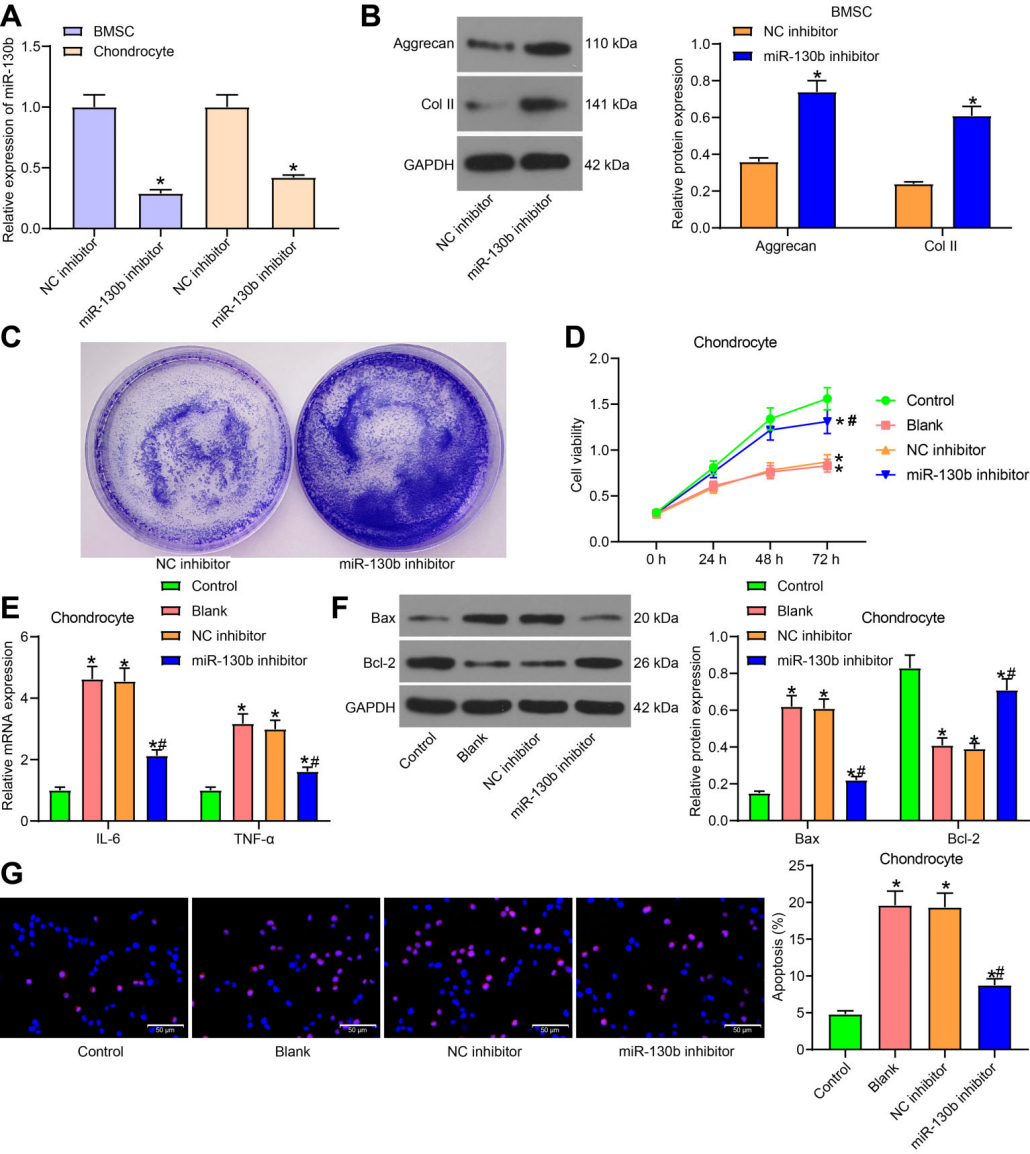
Inhibition of miR-130b promotes chondrogenic differentiation of bone marrow mesenchymal stem cells (BMSCs) and chondrocyte growth. **A**, The transfection efficiency of miR-130b inhibitor was verified by RT-qPCR. **B**, Western blot analysis measured the expression of two cartilage markers in BMSCs. **C**, The synthesis of extracellular matrix during chondrogenesis of BMSCs was examined by toluidine blue staining. **D**, MTT method was used to measure the viability of interleukin (IL)-1β-stimulated chondrocytes. **E**, IL-6 and tumor necrosis factor (TNF)-α expression in IL-1β treated chondrocytes was determined by RT-qPCR. **F**, Levels of apoptosis-related proteins in IL-1β-treated chondrocytes were measured by western blot analysis. **G**, Apoptosis rate in IL-1β-treated chondrocytes was measured by TUNEL assay (scale bar, 50 μm). *P<0.05, compared to control; ^#^P<0.05 compared to the NC inhibitor group. The data in panels A and G were analyzed using one-way ANOVA, and the data in panels B, D, E, and F were analyzed using two-way ANOVA. Experiments were repeated 3 times independently. NC: negative control.

**Figure 4:**
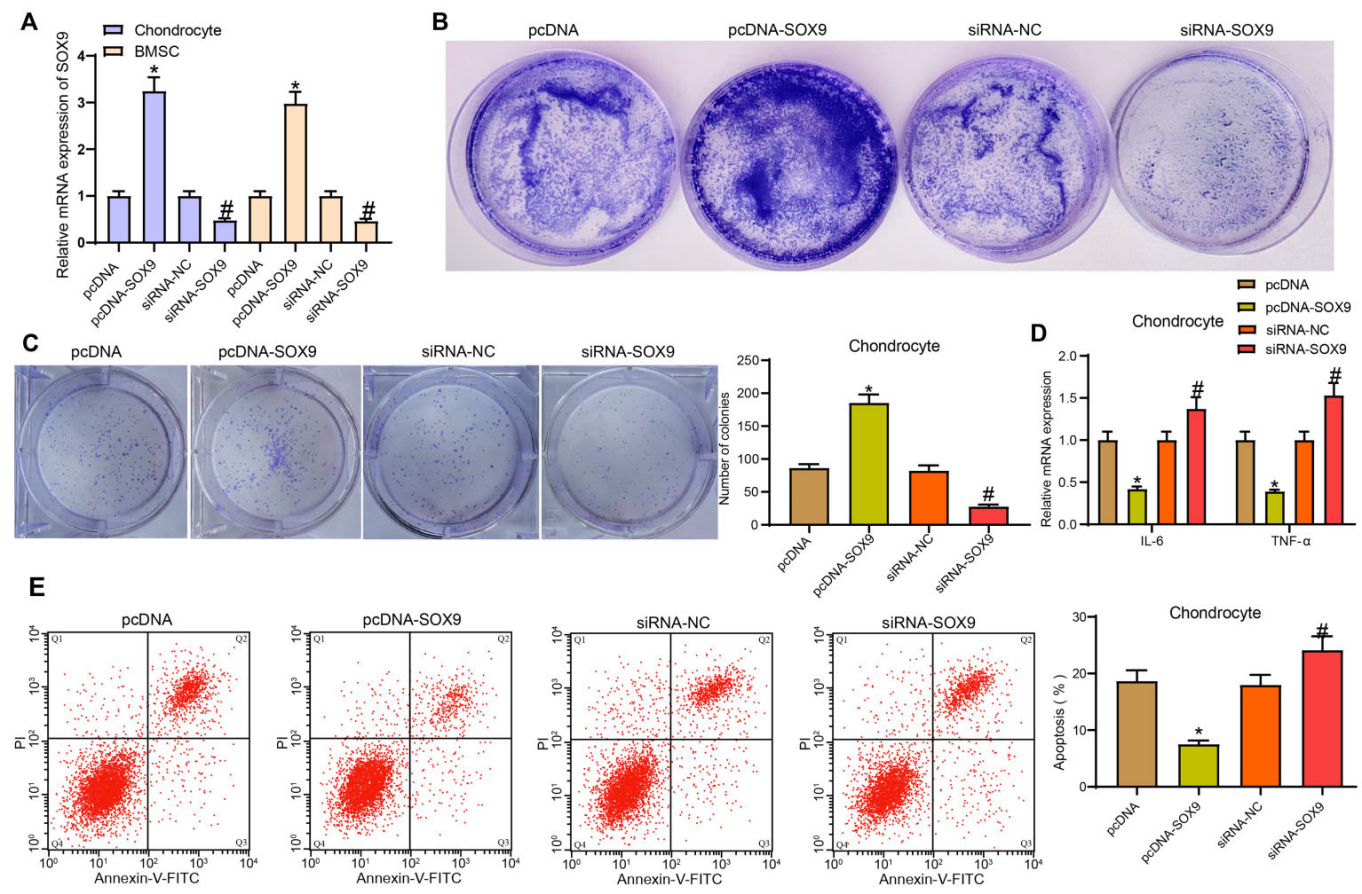
Overexpression of SOX9 promotes chondrogenic differentiation of bone marrow mesenchymal stem cells (BMSCs) and chondrocyte growth. **A**, Transfection efficiency measured by RT-qPCR. **B**, Toluidine blue staining detected the effect of pcDNA-SOX9 and siRNA-SOX9 on the synthesis of extracellular matrix in the process of cartilage differentiation of BMSCs. **C**, Effect of pcDNA-SOX9 and siRNA-SOX9 on the proliferation of interleukin (IL)-1β-treated chondrocytes measured by colony formation assay. **D**, RT-qPCR measured the effects of pcDNA-SOX9 and siRNA-SOX9 on the expression of IL-6 and TNF-α. **E**, Effect of pcDNA-SOX9 and siRNA-SOX9 on apoptosis of IL-1β-treated chondrocytes measured by flow cytometry. The data in panels A, C, and E were processed using one-way ANOVA, and the data in panel D were analyzed using two-way ANOVA. *P<0.05 compared to cells transfected with pcDNA; ^#^P<0.05 compared to cells transfected with siRNA-NC. Experiments were repeated 3 times independently.

**Figure 5:**
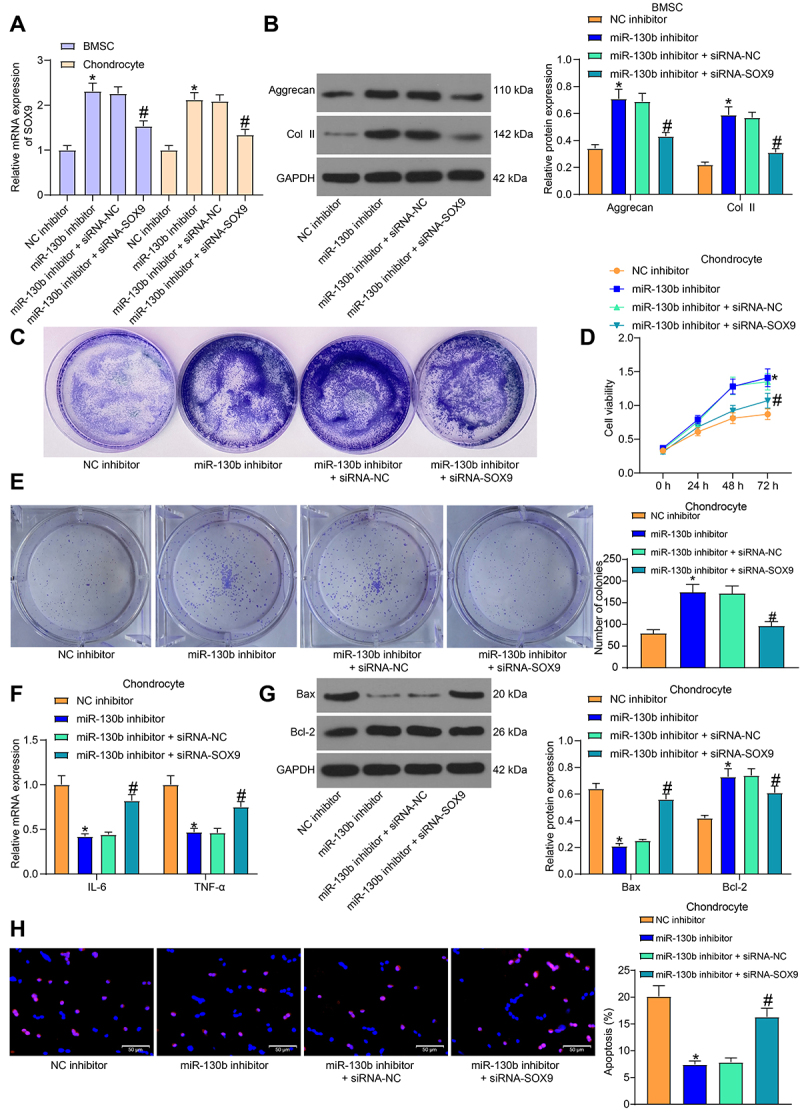
SOX9 silencing weakened the inhibition of miR-130b inhibitor on chondrogenic differentiation of bone marrow mesenchymal stem cells (BMSCs) and chondrocyte growth. **A**, Expression of SOX9 in BMSCs and chondrocytes was measured by RT-qPCR. **B**, Western blot analysis measured the protein level of aggrecan and Col II in BMSCs. **C**, Toluidine blue staining indicated the extracellular matrix synthesis of BMSCs. **D**, MTT method measured the viability of chondrocytes. **E**, Colony formation ability of chondrocytes. **F**, RT-qPCR measured the expression of interleukin (IL)-6 and tumor necrosis factor (TNF)-α in chondrocytes. **G**, Western blot analysis measured the expression of apoptosis-related factors in chondrocytes. **H**, TUNEL staining examined the apoptosis rate of chondrocytes (scale bar, 50 μm). The data in panels A, E, and H were analyzed using one-way ANOVA, and the data in panels B, D, F, and G were analyzed using two-way ANOVA. *P<0.05 compared to cells transfected with NC inhibitor; ^#^P<0.05 compared to cells transfected with miR-130b inhibitor + siRNA-NC. Experiments were repeated 3 times independently. NC: negative control.

